# Antimony: a cryptic metabolism disruptor ubiquitous in food contact materials

**DOI:** 10.1210/jendso/bvag011

**Published:** 2026-01-22

**Authors:** Luyu Wang, Spyridoula Gerassimidou, Birgit Geueke, Ksenia J Groh, Eleni Iacovidou, Aaish Majid, Olwenn Martin, Jane Muncke, Lindsey V Parkinson, Margaret C Weiss, Robert M Sargis

**Affiliations:** College of Medicine, University of Illinois at Chicago, Chicago, IL 60612, USA; Division of Endocrinology, Diabetes, and Metabolism, University of Illinois at Chicago, Chicago, IL 60612, USA; Brunel University of London, Department of Civil and Environmental Engineering, College of Design, Engineering and Physical Sciences, Uxbridge UB8 3PH, UK; Food Packaging Forum Foundation, Zurich CH-8045, Switzerland; Department of Environmental Toxicology, Eawag—Swiss Federal Institute of Aquatic Science and Technology, Duebendorf CH-8600, Switzerland; Brunel University of London, Department of Civil and Environmental Engineering, College of Design, Engineering and Physical Sciences, Uxbridge UB8 3PH, UK; College of Medicine, University of Illinois at Chicago, Chicago, IL 60612, USA; Division of Endocrinology, Diabetes, and Metabolism, University of Illinois at Chicago, Chicago, IL 60612, USA; Plastic Waste Innovation Hub, UCL Arts and Science, University College London, London WC1E 6BT, UK; Food Packaging Forum Foundation, Zurich CH-8045, Switzerland; Food Packaging Forum Foundation, Zurich CH-8045, Switzerland; College of Medicine, University of Illinois at Chicago, Chicago, IL 60612, USA; Division of Endocrinology, Diabetes, and Metabolism, University of Illinois at Chicago, Chicago, IL 60612, USA; School of Public Health, University of Illinois at Chicago, Chicago, IL 60612, USA; College of Medicine, University of Illinois at Chicago, Chicago, IL 60612, USA; Division of Endocrinology, Diabetes, and Metabolism, University of Illinois at Chicago, Chicago, IL 60612, USA; Chicago Center for Health and Environment, Chicago, IL 60612, USA

**Keywords:** antimony, food contact material, plastics, cardiometabolic, polyethylene terephthalate, migration

## Abstract

Antimony (Sb) is a group 15 metalloid that is used as a catalyst in the production of polyethylene terephthalate (PET) plastic, a common food contact material (FCM). PET accounts for over 44% of single-use beverage packaging units and is also used in the production of food trays, storage containers, and other items. Due to its frequent co-occurrence with other metals, Sb is also a common contaminant in crystalware, ceramics, and metal FCMs. In light of the increasing use of Sb-containing FCMs in modern society, a thorough evaluation of Sb's potential effect on public health is warranted. Burgeoning evidence suggests Sb is linked to common cardiometabolic conditions, including dyslipidemia, obesity, diabetes, hypertension, heart failure, and atherosclerotic cardiovascular disease. Thus, this review aims to (1) perform a comprehensive systematic assessment of Sb migration from FCMs into foodstuffs and food simulants, (2) obtain an overview of antimony-related health risks, and (3) inform the generation of harm-reduction guidelines at the individual and systems levels.

Antimony (Sb) is a group 15 metalloid used as a catalyst for producing polyethylene terephthalate (PET). Low concentrations of Sb are dispersed into the plastic matrix to produce rigid food packaging [1]. Over 70 million tons of PET are produced globally per year, with production increasing by ∼4% annually [2, 3]. Numerous studies show that Sb can migrate into packaged foods and beverages over time [4-6]. Indeed, Sb has been shown to be one of the most concentrated metal leachates released from plastic food containers at high temperatures [7]. Thus, PET packaging is likely an important source of oral Sb exposure. Given the widespread use of PET plastics in food and beverage packaging, Sb exposure is a potentially significant public health concern [8].

The growing prevalence of Sb in plastic products means that human exposure to Sb is at unprecedented levels [9]. Sb is a heavy metalloid that is toxic at even low concentrations [10, 11]. Epidemiological and experimental evidence has linked Sb exposure to several adverse health effects, particularly cardiometabolic disorders such as diabetes [12-17], obesity [18, 19], hypertension [20-27], and atherosclerotic cardiovascular disease (ASCVD) [28-31].

More than half a billion people suffer from cardiometabolic conditions [32], with annual economic costs projected to exceed $1 trillion by 2030 [33, 34]. While genetic predisposition, excess energy intake, and insufficient energy expenditure contribute to the rising prevalence of cardiometabolic disorders, these factors fail to fully account for the rising tide of disease. Over the last two decades, environmental toxicants have emerged as factors promoting disease susceptibility [35, 36]. Indeed, over 1000 chemicals have been identified as endocrine-disrupting chemicals (EDCs) [37], with many linked to cardiometabolic dysfunction [38-40]. EDCs are defined by the Endocrine Society as “an exogenous chemical, or mixture of chemicals, that can interfere with any aspect of hormone action” [41]. Burgeoning data now clearly implicate EDCs in cardiometabolic dysfunction through various disruptions in metabolic homeostasis [42]. Consequently, exposed individuals are likely at higher risk of developing cardiometabolic disorders, while disproportionate exposures among vulnerable populations may contribute to health disparities [43-45]. Thus, identifying and reducing exposures to EDCs may reduce overall disease burden and improve health equity. The analysis presented herein focuses on Sb, an underappreciated EDC found in food contact materials (FCMs) and food contact articles (FCAs).

With Sb-containing FCMs becoming ubiquitous in the processing, packaging, storing, and consumption of food and beverage products, a thorough investigation of Sb-associated health risks is warranted. The World Health Organization (WHO) recommends <20 μg Sb/L drinking water and has established a tolerable daily intake of 6 µg Sb/kg body weight [46]; however, region-specific guidelines differ. For example, the European Union (EU) had set their Sb drinking water limit at 5 μg/L, but recently raised this to 10 μg/L [47], whereas the US Environmental Protection Agency (US EPA) recommends <6 μg/L drinking water and <0.35 μg Sb/kg body weight per day for oral exposure [48]. Although US EPA does not currently have a regulatory limit for Sb migration into food, most studies show migration levels below the limits set by WHO (20 ppb; 0.02 mg/kg) and the EU (40 ppb; 0.04 mg/kg). Even so, a majority of studies reporting minimal Sb leaching (<2.5 μg/L) from beverage bottles only tested water under typical storage conditions [49-52]. Leaching is substantially enhanced with increasing acidity, temperature, and storage time, resulting in levels greater than 5 μg/L [4, 52-54]. Despite this marked variability in Sb migration, cumulative contact with Sb-containing FCAs has yet to be evaluated; moreover, the cardiometabolic effects of Sb were not assessed prior to its authorization for use in FCMs. Hence, in order to facilitate determination of Sb's risk to current consumers, the Database on Migrating and Extractable Food Contact Chemicals (FCCmigex) [55] was used to (1) perform a comprehensive assessment of Sb migration from FCMs into foodstuffs and food simulants, (2) obtain an overview of antimony-related health risks, and (3) inform the generation of harm-reduction guidelines at the individual and systems levels.

## Methods

### Antimony-containing food contact articles: literature search

**Figure 1:**
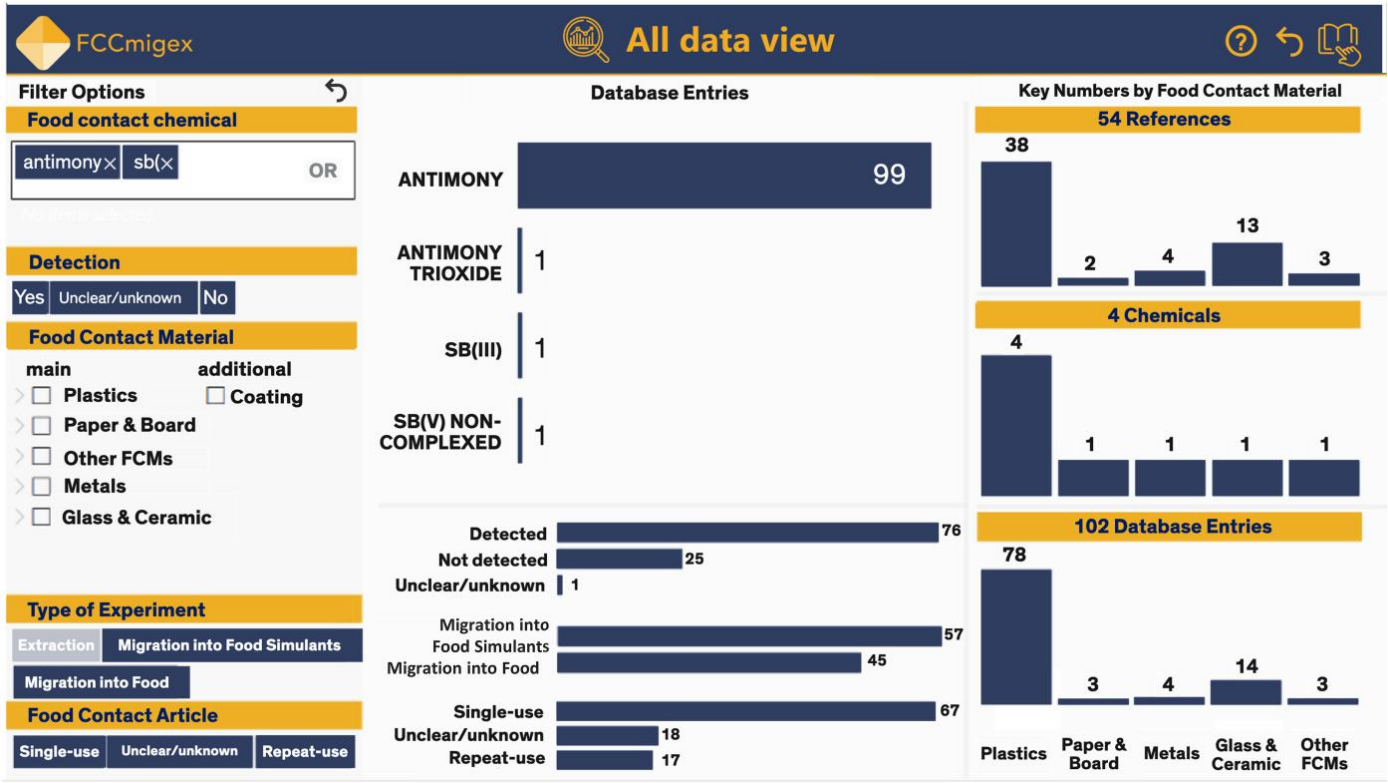
Sb and food packaging: FCCmigex database results. Representative image of the FCCmigex database following filtration for references that analyzed the migration of Sb from all types of FCAs (single-use, unclear/unknown, or repeat-use). This included 102 database entries (78 plastics, 3 paper & board, 14 glass & ceramic, and 3 other FCMs) from 54 references matching filter criteria. The total number of references was reduced to 40 following abstract review for duplicates, non-English articles, and extraction experiments. Figure redrawn in BioRender. Wang, L. (2026) https://BioRender.com/32yo4rf.

Scientific reports analyzing Sb migration from FCAs were retrieved from FCCmigex, version 3 [55]. The database was filtered for references that analyzed the migration of Sb from all types of FCAs where Sb was detected in food and/or food simulants (Fig. 1). Migration is generally considered a proxy for human exposure, as chemicals migrating from FCMs are very likely to be ingested with foodstuffs. In total, 54 references matched filter criteria for Sb migration into food or food simulants. Abstracts were filtered to remove duplicates, non-English publications, and extraction experiments. For all 40 retained references, we mapped experimental details and migration results (eg, duration, temperature, type of food/food simulant, type of FCM, and Sb migration levels).

### Data extraction and compilation of Sb levels

We compiled the following information from the selected studies on Sb migration from FCMs:

- Bibliographic information;
- Chemical form of Sb detected, eg, elemental form (Sb) or antimony oxide form (Sb_2_O_3_);
- Migration limits as specified by EU and US legislation, ie, specific migration limit set by Commission Regulation (EU) No. 10/2011 along with the reference number of the substance as given in the Union list (FCM No.) and allowable levels for bottled water in the US [56] since no migration limits are listed for “substances added to food”;
- Initial concentration of Sb in FCAs before migrating (if available);
- Detected Sb concentration in food/food simulant, including the limit of detection (LOD) expressed in mg/kg;
- Number of analyzed samples for the given migrated concentration—clarifying whether this number referred to replicates from the same sample or to distinct samples;
- Migration conditions and factors that were investigated during migration experiments, eg, storage duration (usually days), storage temperature, ultraviolet radiation exposure, pH, or any other condition applied during migration testing;
- Food simulant or food sample into which Sb was found to migrate;
- Chemical analysis method used to determine migration levels;
- Type of packaging material, eg, PET, metal, glass, crystalware, etc.;
- The source of the food packaging material, ie, whether it was virgin or recycled, including the recycled content (if available);
- The use of PET article, ie, whether it was single-use or repeat-use;
- Reported form of food packaging article, eg, bottle, preform, pellet, container, tray, films, etc., including characteristics such as capacity/color (if available);
- Purchase location of FCA (if available); and
- The lifecycle stage depending on the migration conditions and the source of food packaging material, eg, migration experiments under specified storage conditions refer to the stage of storage and distribution, while migration experiments in virgin food packaging articles that do not consider any storage conditions refer to the stage of production, and migration experiments in recycled food packaging articles refer to the stage of reprocessing.

### Urinary antimony levels in the USA

We assessed the average concentration of urinary Sb in a large nationally representative sample of the US population [57]. Briefly, the National Health and Nutrition Examination Survey (NHANES) is a cross-sectional survey representing the general US population that has been conducted continuously in two-year cycles beginning in 1999. Demographic, dietary, physical examination, laboratory, and questionnaire data are collected from each participant with complex sampling and survey weighting. Utilizing four NHANES cycles from 2011 to 2018, 11 457 participants eligible for urinary metal measurements were assessed for Sb levels. The eligible sample included all examined participants aged 3 to 5 years and a random one-third subsample of participants aged 6 years and older. Accounting for survey design and outliers, we summarized geometric mean and 95% confidence interval for urinary Sb among 10 901 NHANES participants without missing urine measurements. The lower LOD for Sb in urine utilizing mass spectrometry was 0.022 μg/L. Among participants without missing samples, 23.92% of samples were below the LOD and were assigned a concentration of the LOD divided by $\sqrt{2}$. Averages were estimated using SAS software version 9.4 (SAS Institute Inc., Cary, NC).

### Antimony-related health risks: literature search

**Figure 2:**
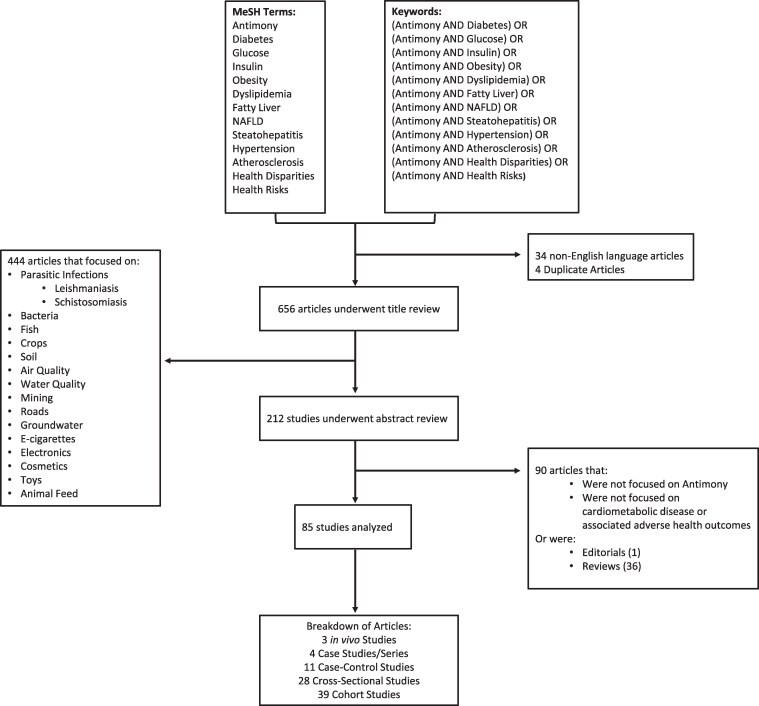
Sb and cardiometabolic disease: Flowchart of systematic literature review in PubMed. Representative chart of MeSH Terms, keywords, and filtering mechanisms utilized to review current literature linking Sb and cardiometabolic health outcomes. The initial search yielded 656 articles, with 444 articles being removed from further review due to their focus on environmental outcomes, toys, cosmetics, or parasitic infections. Of the remaining 212 studies, 53 articles did not directly examine antimony or cardiometabolic disease outcomes, and 37 references were not primary literature. The final 85 articles meeting the inclusion criteria included: 3 in vivo studies, 4 case studies/series, 11 case-control studies, 28 cross-sectional studies, and 39 cohort studies.

Thirteen Medical Subject Heading (MeSH) terms were chosen and combined as described in Fig. 2. This returned 694 PubMed results from 1954 to May 12, 2025. Of these, 34 were non-English articles and 4 were duplicates; these 38 articles were excluded from analysis. The remaining 656 articles underwent title review to determine relevance to cardiometabolic disease. This led to the exclusion of 444 additional articles that were focused on topics outside the scope of this review. The remaining 212 articles were assessed based on abstract content. Of these articles, 127 were excluded for (1) not being focused on Sb, (2) not being focused on cardiometabolic disease and associated comorbidities, and/or (3) being editorials or review articles. This generated a total of 85 articles for analysis. Of these, 3 were *in vivo* studies, 4 were case studies/case reports, 11 were case-control studies, 28 were cross-sectional studies, and 39 were cohort studies.

## Results

### Human biomonitoring for Sb

Utilizing NHANES data from 2011 to 2018, the geometric mean for urine Sb was 0.0473 μg/L (95% CI: 0.0462, 0.0483, LOD: 0.022 μg/L) among 10 901 participants weighted to represent the general U.S. population. This is similar to data reported by the 2009-2011 Canadian Health Measures Survey, for which the geometric mean urinary Sb concentration was 0.048 μg/L (95% CI: 0.046, 0.050) for an analytical sample of 6311 individuals between the ages of 3 and 79 years; in this analysis, 20% of values were below the LOD of 0.02 μg/L [58]. Findings from the 2017-2018 China National Human Biomonitoring Survey (CNHBS) were also comparable: 0.05 μg/L (95% CI: 0.02,0.09) for a population of 11 037 individuals; however, the LOD was higher in this study (0.045 μg/L for urine), with 48.9% of subjects having urinary Sb levels below the LOD [21]. In contrast, blood measurements for the CNHBS population revealed an average Sb concentration of 2.35 μg/L (LOD of 0.03 μg/L). These data are summarized in Table 1. Population-based biomonitoring data could not be found for Japan, but a recent analysis of global DNA methylation in umbilical cord blood showed a consistent positive association with Sb levels [59].

**Table 1:** Human biomonitoring for Sb in urine and blood by country

Country	Limit of detection for Sb (μg/L)	Urinary Sb concentrations (μg/L)	Blood Sb concentrations (μg/L)
USA (NHANES)	0.022	0.0473 (95% CI: 0.0462, 0.0483)	Not available
Canada (CHMS)	0.02	0.048 (95% CI: 0.046, 0.050)	Not available
China (CNHBS)	Urine: 0.045 Blood: 0.03	0.05 (95% CI: 0.02, 0.09)	2.35 (95% CI: 1.92, 2.93)

Compilation of urine and blood biomonitoring for Sb from publicly available data and published literature for the USA, Canada, and China.

### Antimony exposure via food contact articles

All literature cited in this section was collected from the FCCmigex database search for Sb. Sb migration levels vary with changes in temperature, type and acidity of foodstuff, and time [6, 50, 53, 54, 60-77]. Sb leaching from PET also depends on the nature of the Sb migrant (ie, its chemical speciation) and the morphology of the plastic polymer [5]. Migration also increases with greater use of Sb-containing recycled plastic waste in FCAs [78, 79]. Higher baseline Sb levels in FCAs raises concern for increased Sb leaching during the storage of acidic foodstuffs like soft drinks and juices [61, 74, 77] as well as during heating of foodstuffs in Sb-containing plastic containers [75]. Although the potential health risks of Sb exposure from PET bottles have been considered acceptable in most cases [49-51], some samples exceeded established Sb limits even under normal storage conditions [61, 67-69]. Therefore, it is possible that total exposures from PET and other FCMs are higher than the guideline values. Critically, few studies have been performed to assess cumulative Sb exposure from PET and other FCMs. This is of special concern due to the rise in plastic usage and the lack of data for baseline exposure estimates [80, 81].

FCCmigex can be used to fill such data gaps. According to the FCCmigex database (version 3, including data published by May 2025), Sb migration into foods and/or food simulants has been detected from plastics, glass, ceramics, metal, and other/undefined FCMs. A search for “Antimony” and “Sb” on May 12, 2025, returned 54 results. Of the 40 relevant migration papers, 33 examined PET plastics used to generate beverage bottles and ready-made meal trays. In 1995, an early study on Sb migration from plastic FCMs reported Sb levels of 1.1 to 3.9 μg/kg food simulant in migrates from PET samples; however, in migrates of non-PET polymers, the Sb levels were below the LOD [82]. Since then, dozens of migration studies have analyzed Sb migration from PET, but also from glass, ceramics, paper, and metal [75, 83-86]. Following data extraction from the compiled literature, we found that approximately 66% of experiments tested migration into pure or distilled water, while a minority of experiments tested acidified water/beverages (23%) or other consumed food products (11%). Ranges of migration levels are summarized in Table 2 for different FCMs under various experimental conditions. Migration appears to vary by FCM type, as migrates from PET showed higher Sb levels than glass bottles [63, 86]. Regardless of the material, Sb levels below 20 μg/kg food or food simulant were detected in most of the experiments [60, 64, 87, 88]. However, we identified multiple studies in which Sb levels >100 μg/kg food simulant were reached in migrates of inorganic FCMs, such as crystal glass [89] and glazed ceramics [90]. Sb was released from stainless steel sheets into acidic food simulants at varying levels depending on the material's grade, reaching concentrations between <0.1 and 4 μg/kg food simulant [85]. Sb migration levels up to 241, 88, and 38 μg/kg food were observed in doughs prepared in PET baking dishes, meat and fish roasted in PET bags, and ready-to-eat meals heated in PET trays, respectively [5]. A separate study from the same group reported an increase of 1.7 to 14.9 μg Sb per kg food in heated ready-made meals [6]. Collectively, these data indicate that despite high variability in migration due to differences in study conditions, FCMs are a potentially significant source of Sb exposure.

**Table 2:** Summary of Sb migration levels for different FCMs under various experimental conditions

Material	Below LOD, %	Range of Sb migration levels (μg Sb/kg food or food simulant)
Paper	0%; (0/9)	1 × 10^−3^ to 3 × 10^−2^
Plastic	7%; (56/802)	1.1 × 10^−7^ to 241
Glassware	66.7%; (6/9)	4 × 10^−5^ to 2.8 × 10^−2^
Crystalware	8.7%; (4/46)	1.6 × 10^−1^ to 170
Ceramics, Porcelain, Earthenware	29.7%; (11/37)	3.6 × 10^−3^ to 141
Metals	0%; (0/8)	1 × 10^−1^ to 4

The FCCmigex identified Sb in several FCMs. The number of experiments that reported migration levels below the LOD are expressed as a percentage relative to all experiments examining migration levels in each FCM. Minimum and maximum reported migration levels (μg/kg) are listed for each FCM.

### Antimony and cardiometabolic disease

**Figure 3:**
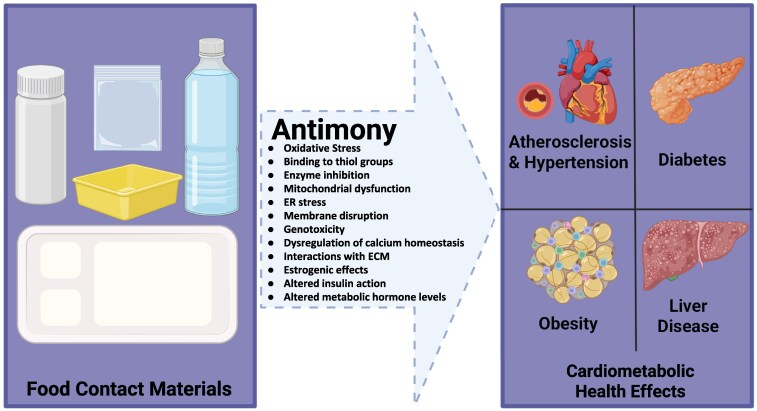
Sb and associated cardiometabolic health risks. Sb is a common catalyst in the manufacturing of PET plastic FCMs, and the current literature links Sb exposure to a variety of adverse health effects, including atherosclerotic cardiovascular disease, hypertension, diabetes, liver disease, and obesity. Potential mechanisms of Sb-mediated toxicity are listed within the dashed arrow. ER, endoplasmic reticulum; ECM, extracellular matrix, ROS, reactive oxygen species. Created in BioRender. Wang, L. (2025) https://BioRender.com/yjlawz8.

Given the increasing use of Sb in FCMs, we proceeded to synthesize the available data on Sb exposure and cardiometabolic outcomes. Epidemiological studies have linked Sb exposure to various health conditions, including impaired fasting glucose, insulin resistance, obesity, hypertension, and dyslipidemia [91]. Although it is difficult to examine the isolated health effects of Sb due to its frequent co-occurrence with other nonessential (“toxic”) metals in mixtures [92], emerging human and animal data indicate a role for Sb in the development of multiple cardiometabolic disorders. A summary of these findings is illustrated in Fig. 3.

#### Diabetes

While five studies showed no significant link [93-95] or even negative associations [96, 97] between Sb and diabetes, a number of other studies indicated that Sb may be diabetogenic. In gestational diabetes mellitus and type 2 diabetes mellitus, elevations in blood glucose stem from insufficient insulin secretion from β-cells, a process that may be induced or amplified by peripheral insulin resistance. Impairments in glucose homeostasis manifest as elevated fasting and postprandial blood glucose levels with accompanying increases in hemoglobin A1c (HbA_1C_) levels, an integrative marker of glycemic status. Sb exposure has been associated with elevated HbA_1C_ in adolescents, particularly males [98]. A cross-sectional study in Wuhan, China, also linked Sb exposure to increased diabetes risk, particularly in the highest quartile of exposure [12]. However, the association was lost after adjustment for anti-hyperglycemic medication use and history of diabetes, suggesting that Sb-mediated effects may be influenced by diabetes status [12], highlighting the need for longitudinal studies stratified by glycemic status. Data from a 1999-2010 NHANES analysis showed positive associations between Sb levels and insulin resistance as assessed by the Homeostatic Model Assessment for Insulin Resistance (HOMA-IR) [13]. Similarly, a 2020 analysis of blood from subjects with polycystic ovary syndrome revealed positive correlations between Sb levels and both fasting plasma glucose and HOMA-IR [99]. Positive associations between Sb and fasting plasma glucose were similarly noted in a cohort of women from the Manganese-Exposed Workers Healthy Cohort in China [17]. Several studies in pregnant women also noted positive associations between Sb and gestational diabetes risk [15, 93, 94, 97]. Supporting this epidemiological data, rodent models suggest that Sb can increase serum glucose levels in female rats at high levels of exposure (60 mg Sb/kg/day for 28 days) [100], though an earlier study indicated that 90 days of exposure to Sb at approximately 0.64, 6.13, and 45.69 mg/kg/day can lower glucose levels in female rats [101]. Importantly, neither rodent study examined dynamic changes to glucose homeostasis or alterations in insulin levels, critical areas for future investigation. Collectively, these data indicate that Sb may have the capacity to disrupt glucose homeostasis, including during sensitive windows of development.

#### Obesity

The relationship between Sb and obesity remains inconclusive. An early analysis of 1999-2002 NHANES data showed no significant relationship between Sb and waist circumference or body mass index [102]. A 1999-2011 NHANES study also found no significant correlation between urinary Sb and obesity in children aged 6 to 19 years [103]. Additionally, data from the Guangdong Provincial Residences’ Chronic Disease and Nutrition Surveillance Survey showed no significant association between Sb exposure and obesity measures [104]. Despite these null findings, a 2003-2014 NHANES study found positive associations between Sb-containing metal mixtures and obesity, hypertension, and type 2 diabetes mellitus; however, that study did not identify Sb exposure alone as a risk factor [19]. A more recent analysis using NHANES 1999-2016 data revealed a curvilinear relationship between urinary Sb levels and obesity, with moderate Sb levels associated with obesity [18]. In a study of childhood cardiometabolic risk, Bayesian Kernel Machine Regression modeling revealed a negative association between Sb and levels of leptin, a hormone critical for promoting satiety; this finding may indicate a mechanism by which Sb can alter body weight regulation [105]. Given the current state of the evidence, longitudinal studies are needed to fully elucidate the impact of Sb exposure on adiposity.

#### Liver disease

Hepatotoxicity is a well-documented side effect of pentavalent antimonial drugs used to treat leishmaniasis, the use of which result in high Sb exposure (∼20 mg Sb/kg/day for 20 to 30 days) [106]. However, hepatic dysfunction has also been observed at lower exposure levels. Epidemiological analyses linked elevated plasma Sb levels (>0.09 μg/L) to increases in total and direct bilirubin in an adult Chinese population [107]. Bilirubin is a by-product of red blood cell (RBC) breakdown that is enzymatically conjugated by hepatocytes and excreted into the bile. Thus, elevations in bilirubin may indicate impairments in hepatic bilirubin metabolism and/or biliary excretion. Complementary to findings of Sb associations with bilirubin levels, a recent NHANES study (2003-2018) demonstrated that urinary Sb (0.03-0.09 μg/L) was positively linked to metabolic dysfunction-associated steatotic liver disease (previously “nonalcoholic fatty liver disease”) [108]. In animal models, a study in rats examining a range of Sb exposures [0, 0.06, 0.6, 6, and 60 mg Sb^3+^/kg/day for 28 days] demonstrated an increase in liver-to-body weight ratio at the highest dose in both sexes, with an increase in relative liver weight also seen in female rats exposed to 6 mg Sb/kg/day [100]. In this same study, liver enzymes were also altered. Alkaline phosphatase (ALP) levels were reduced in male rats at all doses except the 6 mg Sb/kg/day dose, while the aspartate aminotransferase (AST)-to-alanine aminotransferase (ALT) ratio (AST:ALT) was reduced at all doses in female rats. These alterations in liver enzymes suggest a capacity for Sb to induce liver dysfunction, as ALP is an enzyme crucial to nutrient processing in the liver and altered AST:ALT ratios can be indicative of liver damage. This supposition is reinforced by blood metabolomics analyses from these rats indicating dose-dependent disruptions in glucose and lipid metabolism [100]. Evidence of Sb-induced alterations in liver health are further supported by a study of Kunming mice exposed to 15 mg Sb/kg/day for 60 days. Sb-exposed mice exhibited increased liver-to-body weight ratio, elevations in serum ALT, AST, and ALP, and irregular nuclei and chromosome marginalization in hepatocytes [109].

#### Hypertension

Hypertension is a state of chronic blood pressure elevations that is a potent driver of cardiovascular disease. Studies of the 2009-2010 and 2011-2012 NHANES cycles showed a significant association between Sb exposure and higher blood pressure [23, 24]. Evidence from a cohort of children in China showed similar findings, with higher Sb levels positively correlated with systolic blood pressure (SBP), mean arterial pressure (MAP), and odds of having hypertension; moreover, there were significant synergistic interactions between Sb and arsenic (As), another Group 15 metalloid linked to multiple cardiometabolic disorders [22]. In the Alberta Pregnancy Outcomes and Nutrition Cohort, there was a trend toward a positive association between Sb levels and pregnancy-associated hypertension (*P* < .10) [110]. A case-control study in postpartum women in Tehran also revealed that increases in umbilical cord blood Sb levels were associated with a markedly increased risk of preeclampsia [111].

In contrast to this work, data from five other studies showed no significant associations between blood pressure and Sb exposure [20, 21, 112-114]. This heterogeneity across studies could be due to various factors, including lifestyle, potential nonmonotonic dose–response relationships, or complex interactions of toxic metal mixtures. For example, high SBP correlated with low Sb levels, but high cadmium (Cd) and lead (Pb) levels, suggesting a toxicological interaction between the three metals in one study [25]. Experimental studies that test various metal mixtures and Sb exposures are needed to conclusively delineate a role for Sb exposure in the development and/or severity of hypertension.

#### Atherosclerotic cardiovascular disease

ASCVD is caused by the accumulation and modification of lipoproteins that results in the migration and activation of immune and smooth muscle cells in the subendothelial space of arterial walls; these atherosclerotic plaques can be flow-limiting and are susceptible to acute rupture and clot formation, resulting in major adverse cardiovascular events. Sb has been identified as an atherosclerotic risk factor in several studies. Analyses from Spain's Hortega Study and NHANES (1999-2006) support an association between Sb exposure and cardiovascular disease incidence [27, 30, 115]. In addition, scalp hair concentrations of Sb correlated with atherosclerotic plaque severity in the left main coronary artery in a Polish cohort [116]. Results from a Wuhan-Zhuhai cohort in China support these findings, with urinary Sb positively associated with increased 10-year ASCVD risk [28]. Indeed, several studies have positively correlated Sb exposure with increased risk of cardiovascular disease mortality [117-119]. This is further supported by evidence from the Aragon Workers Health Study, which identified Sb as a potential epigenetic modifier and atherosclerotic risk factor in car assembly plant workers [120, 121]. In metal mixture analyses using NHANES data (2003-2016), Sb was shown to be one of the strongest contributors to CVD risk [26]. In the Wuhan-Zhuhai Adult Cohort, Sb was also positively associated with mean platelet volume, which may be an early biomarker for cardiovascular damage [122]. Thus, there is mounting evidence linking Sb exposure to ASCVD risk.

### Molecular mechanisms underlying the health effects of Sb

The mechanisms by which Sb acts are incompletely elucidated; however, there appear to be many parallels with arsenic (As). For example, transcriptional and proteomic profiles of human epidermal keratinocytes exposed to As^3+^ or Sb^3+^ show significant overlap in differentially expressed genes, suggesting similar mechanisms of toxicity [123]. Much like As, Sb toxicity is dependent on its speciation and oxidation state, with trivalent Sb^3+^ compounds showing greater toxicity than pentavalent Sb^5+^ compounds. The pentavalent state predominates under oxidizing to slightly reducing conditions, while the trivalent form predominates under anaerobic conditions and is often the form released during anthropogenic activities. Intracellular reduction of Sb^5+^ compounds is possible [124], either occurring through an enzymatic pathway as observed in Leishmania parasites [125] or nonenzymatically via interaction with small thiols such as trypanothione or glutathione [126]. Sb exposure, especially Sb^3+^, generates reactive oxygen species (ROS) that can overwhelm cellular antioxidant defenses and induce oxidative stress with associated macromolecular damage [127]. Direct interactions may also occur, as Sb can bind thiol (-SH) groups on proteins. Thus, Sb can have diverse effects on biological systems, including DNA damage; inhibition of enzyme activity; membrane disruption; and modulation of energy metabolism, including mitochondrial dysfunction [124, 128, 129]. Sb has been shown to be genotoxic in the bacterial reverse mutation test (Ames test) and in a chromosomal aberration test in cultured mammalian cells [130]. In yeast, Sb^3+^ inhibited DNA double-strand break repair and distorted cytoskeletal architecture [131]. Sb-induced oxidative DNA damage has been noted in zebrafish [132] and in occupationally exposed humans as well [133].

Redox imbalance resulting from oxidative stress [134-136], increased endoplasmic reticulum stress, and disruptions in calcium homeostasis leading to apoptosis [137] have all been highlighted as molecular mechanisms underlying Sb-induced metabolic dysfunction and cardiotoxicity [138]. For example, exposure to Sb^3+^ led to an increase in cardiac calcium currents, which can cause QT prolongation and increase cardiac arrhythmia risk [139]. This effect could be due to Sb's high affinity for sulfhydryl groups [124], which can lead to oxidation of cysteine residues on proteins [129].

Accumulating evidence suggests that Sb also has estrogenic effects, likely occurring through interactions with the estrogen receptor that disrupt normal sex hormone signaling. In human breast cancer cells, the transcriptional profile generated by 1 μM antimony chloride showed a 60.9% match with 1 nM 17-β-estradiol [140]. The relative proliferation effect of antimony on breast cancer cells was also 49.2% that of 10 nM 17-β-estradiol [140]. More recently, it has been shown that Sb^3+^ causes reproductive defects in female zebrafish, including reductions in mature oocytes and a state akin to polycystic ovary syndrome with accompanying ovarian fibrosis [141]. Fish exposed to Sb exhibited elevated levels of gonadotropin-releasing hormone (GnRH), follicle stimulating hormone (FSH), and luteinizing hormone (LH) with a decrease in their estradiol (E2)-to-testosterone (T) (E2:T) ratio [141]. Mechanistically, Sb appeared to activate WNT/β-catenin and TGF-β/Smad pathways to promote extracellular matrix secretion [141]. Given that dysfunction in these pathways is commonly seen in cardiometabolic diseases [142, 143] and that several other estrogenic endocrine disruptors are linked to obesity and related metabolic complications [144], further interrogation of Sb-related cardiometabolic toxicity is warranted. Critically, the proposed molecular mechanisms underlying Sb toxicity are complex and diverse. Indeed, some effects may only occur at higher exposures (eg, with Sb-containing medications), while other negative impacts may be seen at lower levels of exposure relevant to the general population. Identifying Sb's mechanisms of toxicity across exposure groups will be essential for characterizing the broader threats of Sb exposure and for developing intervention strategies to mitigate those risks.

### Environmental disparities and health inequities

Cardiometabolic disorders are a significant driver of morbidity and mortality, and disadvantaged populations are disproportionately affected [145]. An analysis of NHANES data (2005-2006) found a correlation between higher urinary Sb, bisphenol A, and pesticide levels with greater food insecurity in people with diabetes, respiratory, liver, or mental health disorders [146]. These findings may be related to the prevalence of food deserts in lower-income areas that result in greater consumption of packaged foods. Higher exposures may also be a consequence of the localization of industrial activity in low income communities and communities of color [147]. Analysis of pregnant women in the northeastern USA showed that higher nonessential metal exposures were associated with areas of higher crime, greater diversity, lower educational attainment, lower household income, and higher poverty; moreover, Black/Black-Hispanic women had Sb levels that were 35% higher than non-Hispanic White women, while Hispanic women had Sb levels that were 38.3% higher than their non-Hispanic White counterparts [148]. Such exposures in pregnant women pose particular concerns for gestational complications and long-term outcomes in the offspring of women exposed to high levels of Sb. Indeed, evidence from a lower-income Hispanic pregnancy cohort in Los Angeles showed that Sb is a strong predictor of birth weight for gestational age, with moderate to high concentrations associated with lower birth weight [149]. Based on the Developmental Origins of Health and Disease hypothesis that posits early life stressors can lead to long-term adverse health effects [150], these studies suggest that higher developmental exposure to Sb could contribute to a greater lifetime burden of disease in vulnerable communities. Further work is needed to clarify the full impact of Sb exposure disparities on cardiometabolic and other health inequities.

## Perspectives and conclusions

Available migration data for Sb show several conditions under which WHO and EU regulatory limits are exceeded, particularly when the foodstuff is acidic or stored at high temperatures. Sb migration was highest in plastic FCMs, but migration levels were also notably high for ceramics, porcelain, earthenware, and crystalware. In contrast, glassware, paper, and metal showed the lowest migration levels. Collectively, these findings indicate that FCMs represent important yet modifiable sources of Sb exposure for the general population.

Although the epidemiological evidence is somewhat inconsistent, mounting data link Sb to adverse cardiometabolic health. Animal studies further demonstrate that Sb may induce cardiometabolic dysfunction, and mechanistic work strengthens concerns about Sb's molecular toxicity. While additional studies are needed to refine our understanding of exposure sources, clarify mechanisms of action, and establish appropriate regulatory limits, efforts to reduce exposure to known Sb sources is warranted to protect human health under the precautionary principle. While the exact contribution of FCMs to overall Sb exposure remains uncertain, current migration data support prioritizing use of glassware and metal containers over PET plastics. Although paper products show low migration rates, they remain understudied and carry competing exposure risks from organic EDCs like bisphenol A.

To our knowledge, there is currently no established No Observed Adverse Effects Level for human exposure to Sb; however, the Agency for Toxic Substances and Disease Registry Toxicological Profile (ATSDR) cites 0.06 mg Sb/kg/day in rats based on hypoglycemia endpoints [151]. More research is needed to define a No Observed Adverse Effects Level for humans. In the meantime, health professionals should advise reducing consumption of foods packaged in plastics and minimizing the use of plastic storage containers. Such recommendations may be difficult in resource-limited settings overburdened by other social and structural determinants of health, but it remains important to provide harm-reduction strategies whenever possible. Such strategies should include the entire healthcare team (eg, nutritionists, social workers) to help patients identify safer food sources and reduce toxicant exposure.

Further research is needed to clarify links between Sb and cardiometabolic outcomes in longitudinal cohorts. These studies should carefully consider the matrix in which Sb is measured, as a Swedish study found high week-to-week variability in urine Sb, potentially as a consequence of Sb's long biological half-life and differential partitioning into various tissues [151, 152]. Indeed, blood Sb levels in the CNHBS population were substantially higher than urine levels, suggesting blood may be a more accurate measure of systemic Sb levels [21]. Overall, more comprehensive incorporation of different biological matrices (eg, blood, urine, hair, and toenails) as well as Sb speciation [Sb^3+^ vs Sb^5+^] could greatly empower future investigations of this understudied metalloid.

While individual-level interventions matter, addressing environmental health threats requires systems-level solutions. To address Sb-associated health risks, such approaches should include improving PET plastic design, promoting design-for-recycling principles, replacing Sb as a catalyst with safer alternatives, and reducing Sb contamination in recycled plastics while minimizing health and safety tradeoffs and avoiding regrettable substitutions. Furthermore, biomonitoring initiatives should be strengthened with attention to the full spectrum of Sb-associated adverse health effects (cardiometabolic toxicity, endocrine disruption, cancer, etc.). Collectively, such comprehensive efforts could substantially enhance risk assessment while also providing solutions that ultimately reduce population-level Sb exposure. Given antimony's emergence as a cardiometabolic toxicant prevalent in FCMs, such coordinated action is needed to protect human health and promote health equity.

## Data Availability

Data sharing is not applicable to this article as no datasets were generated or analyzed during the current study.

## References

[1] Filella M . Antimony and PET bottles: checking facts. Chemosphere. 2020;261:127732. 32739689 10.1016/j.chemosphere.2020.127732

[2] Diao J, Hu Y, Tian Y, Carr R, Moon TS. Upcycling of poly(ethylene terephthalate) to produce high-value bio-products. Cell Rep. 2023;42(1):111908. 36640302 10.1016/j.celrep.2022.111908

[3] Gardarin Aurelie . LIFE 3.0—LIFE Project Public Page. Accessed May 12, 2025. https://webgate.ec.europa.eu/life/publicWebsite/project/LIFE20-ENV-FR-000596/towards-a-true-circular-economy-of-pet-plastics-and-textiles-thanks-to-enzymatic-recycling-of-waste

[4] Westerhoff P, Prapaipong P, Shock E, Hillaireau A. Antimony leaching from polyethylene terephthalate (PET) plastic used for bottled drinking water. Water Res. 2008;42(3):551‐556. 17707454 10.1016/j.watres.2007.07.048

[5] Haldimann M, Blanc A, Dudler V. Exposure to antimony from polyethylene terephthalate (PET) trays used in ready-to-eat meals. Food Addit Contam. 2007;24(8):860‐868. 17613073 10.1080/02652030701297511

[6] Haldimann M, Alt A, Blanc A, Brunner K, Sager F, Dudler V. Migration of antimony from PET trays into food simulant and food: determination of Arrhenius parameters and comparison of predicted and measured migration data. Food Addit Contam Part A Chem Anal Control Expo Risk Assess. 2013;30(3):587‐598. 23286325 10.1080/19440049.2012.751631 PMC3613973

[7] Zeng X, Liu D, Wu Y, et al Heavy metal risk of disposable food containers on human health. Ecotoxicol Environ Saf. 2023;255:114797. 36933486 10.1016/j.ecoenv.2023.114797

[8] Sax L . Polyethylene terephthalate may yield endocrine disruptors. Environ Health Perspect. 2010;118(4):445‐448. 20368129 10.1289/ehp.0901253 PMC2854718

[9] Nishad PA, Bhaskarapillai A. Antimony, a pollutant of emerging concern: a review on industrial sources and remediation technologies. Chemosphere. 2021;277:130252. 33780676 10.1016/j.chemosphere.2021.130252

[10] Periferakis A, Caruntu A, Periferakis A-T, et al Availability, toxicology and medical significance of antimony. Int J Environ Res Public Health. 2022;19(8):4669. 35457536 10.3390/ijerph19084669 PMC9030621

[11] Fu Z, Xi S. The effects of heavy metals on human metabolism. Toxicol Mech Methods. 2020;30(3):167‐176. 31818169 10.1080/15376516.2019.1701594

[12] Feng W, Cui X, Liu B, et al Association of urinary metal profiles with altered glucose levels and diabetes risk: a population-based study in China. PLoS One. 2015;10(4):e0123742. 25874871 10.1371/journal.pone.0123742 PMC4395404

[13] Menke A, Guallar E, Cowie CC. Metals in urine and diabetes in U.S. Adults. Diabetes. 2016;65(1):164‐171. 26542316 10.2337/db15-0316 PMC4686948

[14] Zhang G, Wang X, Zhang X, et al Antimony in urine during early pregnancy correlates with increased risk of gestational diabetes mellitus: a prospective cohort study. Environ Int. 2019;123:164‐170. 30529888 10.1016/j.envint.2018.11.072

[15] Onat T, Demir Caltekin M, Turksoy VA, et al The relationship between heavy metal exposure, trace element level, and monocyte to HDL cholesterol ratio with gestational diabetes Mellitus. Biol Trace Elem Res. 2021;199(4):1306‐1315. 33219922 10.1007/s12011-020-02499-9

[16] Xiao L, Zhou Y, Ma J, et al Oxidative DNA damage mediates the association between urinary metals and prevalence of type 2 diabetes mellitus in Chinese adults. Sci Total Environ. 2018;627:1327‐1333. 30857096 10.1016/j.scitotenv.2018.01.317

[17] Ge X, Yang A, Huang S, et al Sex-specific associations of plasma metals and metal mixtures with glucose metabolism: an occupational population-based study in China. Sci Total Environ. 2021;760:143906. 33341635 10.1016/j.scitotenv.2020.143906

[18] Swayze S, Rotondi M, Kuk JL. The associations between blood and urinary concentrations of metal metabolites, obesity, hypertension, type 2 diabetes, and dyslipidemia among US adults: NHANES 1999-2016. J Environ Public Health. 2021;2021:2358060. 34733334 10.1155/2021/2358060 PMC8560296

[19] Wang X, Mukherjee B, Park SK. Associations of cumulative exposure to heavy metal mixtures with obesity and its comorbidities among U.S. adults in NHANES 2003-2014. Environ Int. 2018;121:683‐694. 30316184 10.1016/j.envint.2018.09.035 PMC6268112

[20] Xu J, White AJ, Niehoff NM, O’Brien KM, Sandler DP. Airborne metals exposure and risk of hypertension in the Sister Study. Environ Res. 2020;191:110144. 32898563 10.1016/j.envres.2020.110144 PMC7658027

[21] Qu Y, Lv Y, Ji S, et al Effect of exposures to mixtures of lead and various metals on hypertension, pre-hypertension, and blood pressure: a cross-sectional study from the China National Human Biomonitoring. Environ Pollut. 2022;299:118864. 35063540 10.1016/j.envpol.2022.118864

[22] Liu M, Li M, Guo W, et al Co-exposure to priority-controlled metals mixture and blood pressure in Chinese children from two panel studies. Environ Pollut. 2022;306:119388. 35526645 10.1016/j.envpol.2022.119388

[23] Shiue I . Higher urinary heavy metal, arsenic, and phthalate concentrations in people with high blood pressure: US NHANES, 2009-2010. Blood Press. 2014;23(6):363‐369. 24945898 10.3109/08037051.2014.925228

[24] Shiue I . Higher urinary heavy metal, phthalate, and arsenic but not parabens concentrations in people with high blood pressure, U.S. NHANES, 2011-2012. Int J Environ Res Public Health. 2014;11(6):5989‐5999. 24905244 10.3390/ijerph110605989 PMC4078560

[25] Everson TM, Niedzwiecki MM, Toth D, et al Metal biomarker mixtures and blood pressure in the United States: cross-sectional findings from the 1999-2006 National Health and Nutrition Examination Survey (NHANES). Environ Health. 2021;20(1):15. 33583418 10.1186/s12940-021-00695-1 PMC7883578

[26] Guo X, Li N, Wang H, et al Combined exposure to multiple metals on cardiovascular disease in NHANES under five statistical models. Environ Res. 2022;215:114435. 36174761 10.1016/j.envres.2022.114435

[27] Domingo-Relloso A, Grau-Perez M, Briongos-Figuero L, et al The association of urine metals and metal mixtures with cardiovascular incidence in an adult population from Spain: the Hortega Follow-Up Study. Int J Epidemiol. 2019;48(6):1839‐1849. 31329884 10.1093/ije/dyz061 PMC6929535

[28] Zhu C, Wang B, Xiao L, et al Mean platelet volume mediated the relationships between heavy metals exposure and atherosclerotic cardiovascular disease risk: a community-based study. Eur J Prev Cardiol. 2020;27(8):830‐839. 30776917 10.1177/2047487319830536

[29] Navas-Acien A, Silbergeld EK, Sharrett AR, et al Metals in urine and peripheral arterial disease. Environ Health Perspect. 2005;113(2):164‐169. 15687053 10.1289/ehp.7329 PMC1277859

[30] Agarwal S, Zaman T, Tuzcu EM, Kapadia SR. Heavy metals and cardiovascular disease: results from the National Health and Nutrition Examination Survey (NHANES) 1999-2006. Angiology. 2011;62(5):422‐429. 21421632 10.1177/0003319710395562

[31] El-Kersh K, Danielle Hopkins C, Wu X, et al Plasma level of antimony correlates with pulmonary arterial hypertension severity. Curr Res Toxicol. 2022;3:100080. 35800661 10.1016/j.crtox.2022.100080 PMC9254336

[32] Murray CJL . The global burden of disease study at 30 years. Nat Med. 2022;28(10):2019‐2026. 36216939 10.1038/s41591-022-01990-1

[33] Benjamin EJ, Virani SS, Callaway CW, et al Heart Disease and Stroke Statistics—2018 update: a report from the American Heart Association. Circulation. 2018;137(12):e67‐e492. 29386200 10.1161/CIR.0000000000000558

[34] Shah CH, Fonarow GC, Echouffo-Tcheugui JB. Trends in direct health care costs among US adults with atherosclerotic cardiovascular disease with and without diabetes. Cardiovasc Diabetol. 2024;23(1):238. 38978114 10.1186/s12933-024-02324-w PMC11232126

[35] Schulz MC, Sargis RM. Inappropriately sweet: environmental endocrine-disrupting chemicals and the diabetes pandemic. Adv Pharmacol. 2021;92:419‐456. 34452693 10.1016/bs.apha.2021.04.002 PMC8714029

[36] Kirkley AG, Sargis RM. Environmental endocrine disruption of energy metabolism and cardiovascular risk. Curr Diab Rep. 2014;14(6):494. 24756343 10.1007/s11892-014-0494-0 PMC4067479

[37] National Institute of Environmental Health Sciences . *Endocrine Disruptors*. Accessed August 23, 2023. https://www.niehs.nih.gov/health/topics/agents/endocrine/index.cfm

[38] Zamora Z, Wang S, Chen Y-W, Diamante G, Yang X. Systematic transcriptome-wide meta-analysis across endocrine disrupting chemicals reveals shared and unique liver pathways, gene networks, and disease associations. Environ Int. 2024;183:108339. 38043319 10.1016/j.envint.2023.108339 PMC11216742

[39] Lu X, Xie T, van Faassen M, et al Effects of endocrine disrupting chemicals and their interactions with genetic risk scores on cardiometabolic traits. Sci Total Environ. 2024;914:169972. 38211872 10.1016/j.scitotenv.2024.169972

[40] Pestana D, Teixeira D, Sá C, et al The role of endocrine disruptors on metabolic dysfunction. Open Biotechnol J. 2016;10(1):108‐121.

[41] Zoeller RT, Brown TR, Doan LL, et al Endocrine-disrupting chemicals and public health protection: a statement of principles from The Endocrine Society. Endocrinology. 2012;153(9):4097‐4110. 22733974 10.1210/en.2012-1422 PMC3423612

[42] La Merrill MA, Smith MT, McHale CM, et al Consensus on the key characteristics of metabolism disruptors. Nat Rev Endocrinol. 2025;21(4):245‐261. 39613954 10.1038/s41574-024-01059-8 PMC11916920

[43] Ruiz D, Becerra M, Jagai JS, Ard K, Sargis RM. Disparities in environmental exposures to endocrine-disrupting chemicals and diabetes risk in vulnerable populations. Diabetes Care. 2018;41(1):193‐205. 29142003 10.2337/dc16-2765 PMC5741159

[44] Weiss MC, Wang L, Sargis RM. Hormonal injustice: environmental toxicants as drivers of endocrine health disparities. Endocrinol Metab Clin North Am. 2023;52(4):719‐736. 37865484 10.1016/j.ecl.2023.05.009 PMC10929240

[45] Wang L, Flores J, Sargis RM. Disparities in endocrine-disrupting chemical exposures and thyroid disorders. Curr Opin Endocr Metab Res. 2025;41:100591.

[46] World Health Organization. *Guidelines for Drinking-water Quality, 4th edition, Incorporating the 1st Addendum*. Accessed August 17, 2023. https://www.who.int/publications-detail-redirect/9789241549950

[47] Dettori M, Arghittu A, Deiana G, Castiglia P, Azara A. The revised European Directive 2020/2184 on the quality of water intended for human consumption. A step forward in risk assessment, consumer safety and informative communication. Environ Res. 2022;209:112773. 35065937 10.1016/j.envres.2022.112773

[48] United States Environmental Protection Agency. System of Registries | US EPA. Accessed August 17, 2023. https://sor.epa.gov/sor_internet/registry/substreg/searchandretrieve/advancedsearch/externalSearch.do?p_type=CASNO&p_value=7440-36-0

[49] Sánchez-Martínez M, Pérez-Corona T, Cámara C, Madrid Y. Migration of antimony from PET containers into regulated EU food simulants. Food Chem. 2013;141(2):816‐822. 23790852 10.1016/j.foodchem.2013.03.067

[50] Qiao F, Lei K, Li Z, et al Effects of storage temperature and time of antimony release from PET bottles into drinking water in China. Environ Sci Pollut Res. 2018;25(2):1388‐1393. 10.1007/s11356-017-0598-6 29090435

[51] Welle F, Franz R. Migration of antimony from PET bottles into beverages: determination of the activation energy of diffusion and migration modelling compared with literature data. Food Addit Contam Part A Chem Anal Control Expo Risk Assess. 2011;28(1):115‐126. 21184310 10.1080/19440049.2010.530296

[52] Al-Otoum F, Al-Ghouti MA, Costa OS, Khraisheh M. Impact of temperature and storage time on the migration of antimony from polyethylene terephthalate (PET) containers into bottled water in Qatar. Environ Monit Assess. 2017;189(12):631. 29129001 10.1007/s10661-017-6342-3

[53] Keresztes S, Tatár E, Mihucz VG, Virág I, Majdik C, Záray G. Leaching of antimony from polyethylene terephthalate (PET) bottles into mineral water. Sci Total Environ. 2009;407(16):4731‐4735. 19467696 10.1016/j.scitotenv.2009.04.025

[54] Xu S, Zhou P, Li H, Juhasz A, Cui X. Leaching and in vivo bioavailability of antimony in PET bottled beverages. Environ Sci Technol. 2021;55(22):15227‐15235. 34738794 10.1021/acs.est.1c02818

[55] Geueke B, Groh KJ, Maffini MV, et al Systematic evidence on migrating and extractable food contact chemicals: most chemicals detected in food contact materials are not listed for use. Crit Rev Food Sci Nutr. 2023;63(28):9425‐9435. 35585831 10.1080/10408398.2022.2067828

[56] United States Food and Drug Administration. *CFR—Code of Federal Regulations Title 21*. Accessed August 29, 2023. https://www.accessdata.fda.gov/scripts/cdrh/cfdocs/cfcfr/cfrsearch.cfm?fr=165.110

[57] United States Center for Disease Control. *NHANES National Health and Nutrition Examination Survey Homepage*. 2023. Accessed April 6 2023. https://wwwn.cdc.gov/nchs/nhanes/Default.aspx

[58] Government of Canada . *Second Report on Human Biomonitoring of Environmental Chemicals in Canada—Tables 8.1.1 to 8.19.9*. 2016. Accessed April 24, 2023. https://www.canada.ca/en/health-canada/services/environmental-workplace-health/reports-publications/environmental-contaminants/second-report-human-biomonitoring-environmental-chemicals-canada-tables-8-1-1-8-19-9.html

[59] Okamoto Y, Iwai-Shimada M, Nakai K, et al Global DNA methylation in cord blood as a biomarker for prenatal lead and antimony exposures. Toxics. 2022;10(4):157. 35448418 10.3390/toxics10040157 PMC9027623

[60] Fan Y-Y, Zheng J-L, Ren J-H, Luo J, Cui X-Y, Ma LQ. Effects of storage temperature and duration on release of antimony and bisphenol A from polyethylene terephthalate drinking water bottles of China. Environ Pollut. 2014;192:113‐120. 24907857 10.1016/j.envpol.2014.05.012

[61] Tukur A, Sharp L, Stern B, Tizaoui C, Benkreira H. PET bottle use patterns and antimony migration into bottled water and soft drinks: the case of British and Nigerian bottles. J Environ Monit. 2012;14(4):1237‐1247. 22402759 10.1039/c2em10917d

[62] Carneado S, Hernández-Nataren E, López-Sánchez JF, Sahuquillo A. Migration of antimony from polyethylene terephthalate used in mineral water bottles. Food Chem. 2015;166:544‐550. 25053092 10.1016/j.foodchem.2014.06.041

[63] Rowell C, Kuiper N, Preud’Homme H. Is container type the biggest predictor of trace element and BPA leaching from drinking water bottles? Food Chem. 2016;202:88‐93. 26920269 10.1016/j.foodchem.2016.01.109

[64] Cheng X, Shi H, Adams CD, Ma Y. Assessment of metal contaminations leaching out from recycling plastic bottles upon treatments. Environ Sci Pollut Res Int. 2010;17(7):1323‐1330. 20309737 10.1007/s11356-010-0312-4

[65] Molaee Aghaee E, Alimohammadi M, Nabizadeh R, et al Effects of storage time and temperature on the antimony and some trace element release from polyethylene terephthalate (PET) into the bottled drinking water. J Environ Health Sci Eng. 2014;12(1):133. 25431656 10.1186/s40201-014-0133-3 PMC4245802

[66] Bach C, Dauchy X, Severin I, Munoz J-F, Etienne S, Chagnon M-C. Effect of temperature on the release of intentionally and non-intentionally added substances from polyethylene terephthalate (PET) bottles into water: chemical analysis and potential toxicity. Food Chem. 2013;139(1-4):672‐680. 23561160 10.1016/j.foodchem.2013.01.046

[67] Bach C, Dauchy X, Severin I, Munoz J-F, Etienne S, Chagnon M-C. Effect of sunlight exposure on the release of intentionally and/or non-intentionally added substances from polyethylene terephthalate (PET) bottles into water: chemical analysis and in vitro toxicity. Food Chem. 2014;162:63‐71. 24874358 10.1016/j.foodchem.2014.04.020

[68] Mattiazzi P, Bohrer D, Viana C, do Nascimento PC, Veiga M, de Carvalho LM. Determination of antimony in pharmaceutical formulations and beverages using high-resolution Continuum-source graphite furnace atomic absorption spectrometry. J AOAC Int. 2017;100(3):737‐743. 28105980 10.5740/jaoacint.16-0389

[69] Chapa-Martínez CA, Hinojosa-Reyes L, Hernández-Ramírez A, Ruiz-Ruiz E, Maya-Treviño L, Guzmán-Mar JL. An evaluation of the migration of antimony from polyethylene terephthalate (PET) plastic used for bottled drinking water. Sci Total Environ. 2016;565:511‐518. 27192700 10.1016/j.scitotenv.2016.04.184

[70] Allafi AR . The effect of temperature and storage time on the migration of antimony from polyethylene terephthalate (PET) into commercial bottled water in Kuwait. Acta Biomed. 2020;91(4):e2020105. 33525286 10.23750/abm.v91i4.8463 PMC7927525

[71] Shotyk W, Krachler M, Chen B. Contamination of Canadian and European bottled waters with antimony from PET containers. J Environ Monit. 2006;8(2):288‐292. 16470261 10.1039/b517844b

[72] Greifenstein M, White DW, Stubner A, Hout J, Whelton AJ. Impact of temperature and storage duration on the chemical and odor quality of military packaged water in polyethylene terephthalate bottles. Sci Total Environ. 2013;456–457:376‐383. 10.1016/j.scitotenv.2013.03.092 23624011

[73] Shotyk W, Krachler M. Contamination of bottled waters with antimony leaching from polyethylene terephthalate (PET) increases upon storage. Environ Sci Technol. 2007;41(5):1560‐1563. 17396641 10.1021/es061511+

[74] Carneado S, López-Sánchez JF, Sahuquillo Á. Antimony in polyethylene terephthalate-bottled beverages: the migration puzzle. Molecules. 2023;28(20):7166. 37894645 10.3390/molecules28207166 PMC10609323

[75] Han Y, Ryu K, Song N, et al Potential migration and health risks of heavy metals and metalloids in take-out food containers in South Korea. Int J Environ Res Public Health. 2024;21(2):139. 38397630 10.3390/ijerph21020139 PMC10887885

[76] Eti SA, Islam MS, Shourove JH, et al Assessment of heavy metals migrated from food contact plastic packaging: Bangladesh perspective. Heliyon. 2023;9(9):e19667. 37809622 10.1016/j.heliyon.2023.e19667 PMC10558900

[77] Kljaković-Gašpić Z, Tariba Lovaković B, Smoljo I, et al Metal(loid)s, phthalate esters and polycyclic aromatic hydrocarbons in Croatian natural mineral waters: regulatory compliance and associated health risk. Environ Technol Innov. 2024;34:103570.

[78] Gerassimidou S, Lanska P, Hahladakis JN, et al Unpacking the complexity of the PET drink bottles value chain: a chemicals perspective. J Hazard Mater. 2022;430:128410. 35295000 10.1016/j.jhazmat.2022.128410

[79] Turner A, Filella M. Field-portable-XRF reveals the ubiquity of antimony in plastic consumer products. Sci Total Environ. 2017;584–585:982‐989. 10.1016/j.scitotenv.2017.01.149 28190576

[80] Zhu L, Wang ZT, Xu HB, Sun RB, Zhang H, Zhang JB. Exposure assessment of Sb2O3 in PET food contact materials. Biomed Environ Sci. 2016;29(4):305‐313. 27241743 10.3967/bes2016.040

[81] Makris KC, Andra SS, Herrick L, Christophi CA, Snyder SA, Hauser R. Association of drinking-water source and use characteristics with urinary antimony concentrations. J Expo Sci Environ Epidemiol. 2013;23(2):120‐127. 23188481 10.1038/jes.2012.104

[82] Fordham PJ, Gramshaw JW, Crews HM, Castle L. Element residues in food contact plastics and their migration into food simulants, measured by inductively-coupled plasma-mass spectrometry. Food Addit Contam. 1995;12(5):651‐669. 8522030 10.1080/02652039509374354

[83] German Federal Institute for Risk Assessment. *Freisetzung von Metallen aus emaillierten Grillrosten: Einige geben zu viel ab*. Accessed August 29, 2023. https://www.bfr.bund.de/cm/343/freisetzung-von-metallen-aus-emaillierten-grillrosten-einige-geben-zu-viel-ab.pdf

[84] Yang D, Zhu X, Gao J, Song Y, Sui H, Zhu L. Comparison of the test conditions of China and the council of Europe on the release levels of metals from stainless-steel products from the Chinese market. Food Packag Shelf Life. 2022;33:100888.

[85] Qiu K, Yang D, Zhu X, Sui H, Wu G. Survey of six metal contaminants and impurities and eleven metals and alloy components released from stainless-steel sheets on the Chinese market. Food Addit Contam Part A. 2021;38(12):2091‐2101. 10.1080/19440049.2021.1964700 34415827

[86] Reimann C, Birke M, Filzmoser P. Bottled drinking water: water contamination from bottle materials (glass, hard PET, soft PET), the influence of colour and acidification. Applied Geochemistry. 2010;25(7):1030‐1046.

[87] Simoneau C, Beldi G, Peltzer MA, Jakubowska N. Towards suitable tests for the migration of metals from ceramic and crystal tableware: Work in support of the revision of the Ceramic Directive 84/500/EEC. JRC Publications Repository. 2017. https://publications.jrc.ec.europa.eu/repository/handle/JRC108092. doi:10.2760/54169

[88] Zmit B, Belhaneche-Bensemra N. Antimony leaching from PET plastic into bottled water in Algerian market. Environ Monit Assess. 2019;191(12):749. 31728744 10.1007/s10661-019-7891-4

[89] Food Standards Agency. *Investigation of the Significant Factors in Elemental Migration from Glass in Contact With Food*. UK Food Standards Agency Final Report Project Code A03029; 2002. Accessed October 8, 2025.

[90] Demont M, Boutakhrit K, Fekete V, Bolle F, Van Loco J. Migration of 18 trace elements from ceramic food contact material: influence of pigment, pH, nature of acid and temperature. Food Chem Toxicol. 2012;50(3-4):734‐743. 22265939 10.1016/j.fct.2011.12.043

[91] American Diabetes Association . Diagnosis and classification of Diabetes Mellitus. Diabetes Care. 2013;37:S81‐S90. 10.2337/dc14-S081 24357215

[92] Wang X, Karvonen-Gutierrez CA, Herman WH, Mukherjee B, Park SK. Metals and risk of incident metabolic syndrome in a prospective cohort of midlife women in the United States. Environ Res. 2022;210:112976. 35202625 10.1016/j.envres.2022.112976 PMC9869389

[93] Wang X, Gao D, Zhang G, et al Exposure to multiple metals in early pregnancy and gestational diabetes mellitus: a prospective cohort study. Environ Int. 2020;135:105370. 31864020 10.1016/j.envint.2019.105370

[94] Wang Y, Zhang P, Chen X, et al Multiple metal concentrations and gestational diabetes mellitus in Taiyuan, China. Chemosphere. 2019;237:124412. 31376695 10.1016/j.chemosphere.2019.124412

[95] Wang X, Karvonen-Gutierrez CA, Herman WH, Mukherjee B, Harlow SD, Park SK. Urinary metals and incident diabetes in midlife women: Study of Women’s Health Across the Nation (SWAN). BMJ Open Diabetes Res Care. 2020;8(1):e001233. 10.1136/bmjdrc-2020-001233 PMC7398092 32747380

[96] Yuan Y, Xiao Y, Yu Y, et al Associations of multiple plasma metals with incident type 2 diabetes in Chinese adults: The Dongfeng-Tongji Cohort. Environ Pollut. 2018;237:917‐925. 29429611 10.1016/j.envpol.2018.01.046

[97] Zhang Q, Li X, Liu X, et al Association between maternal antimony exposure and risk of gestational diabetes mellitus: a birth cohort study. Chemosphere. 2020;246:125732. 31927364 10.1016/j.chemosphere.2019.125732

[98] Feng B, Tang P, He S, et al Associations between antimony exposure and glycated hemoglobin levels in adolescents aged 12-19 years: results from the NHANES 2013-2016. Front Public Health. 2024;12:1439034. 39484344 10.3389/fpubh.2024.1439034 PMC11524935

[99] Kirmizi DA, Baser E, Turksoy VA, Kara M, Yalvac ES, Gocmen AY. Are heavy metal exposure and trace element levels related to metabolic and endocrine problems in polycystic ovary syndrome? Biol Trace Elem Res. 2020;198(1):77‐86. 32504400 10.1007/s12011-020-02220-w

[100] Gu W, Pang R, Chen Y, et al Short-term exposure to antimony induces hepatotoxicity and metabolic remodeling in rats. Ecotoxicol Environ Saf. 2023;256:114852. 37023648 10.1016/j.ecoenv.2023.114852

[101] Poon R, Chu I, Lecavalier P, et al Effects of antimony on rats following 90-day exposure via drinking water. Food Chem Toxicol. 1998;36(1):21‐35. 9487361 10.1016/s0278-6915(97)80120-2

[102] Padilla MA, Elobeid M, Ruden DM, Allison DB. An examination of the association of selected toxic metals with total and central obesity indices: NHANES 99-02. Int J Environ Res Public Health. 2010;7(9):3332‐3347. 20948927 10.3390/ijerph7093332 PMC2954548

[103] Shao W, Liu Q, He X, Liu H, Gu A, Jiang Z. Association between level of urinary trace heavy metals and obesity among children aged 6-19 years: NHANES 1999-2011. Environ Sci Pollut Res Int. 2017;24(12):11573‐11581. 28321702 10.1007/s11356-017-8803-1

[104] Shen T, Zhong L, Ji G, et al Associations between metal(loid) exposure with overweight and obesity and abdominal obesity in the general population: a cross-sectional study in China. Chemosphere. 2024;350:140963. 38114022 10.1016/j.chemosphere.2023.140963

[105] Kupsco A, Kioumourtzoglou M-A, Just AC, et al Prenatal metal concentrations and childhood cardiometabolic risk using Bayesian Kernel machine regression to assess mixture and interaction effects. Epidemiology. 2019;30(2):263‐273. 30720588 10.1097/EDE.0000000000000962 PMC6402346

[106] Kato KC, Morais-Teixeira E, Reis PG, et al Hepatotoxicity of pentavalent antimonial drug: possible role of residual Sb(III) and protective effect of ascorbic acid. Antimicrob Agents Chemother. 2014;58(1):481‐488. 24189251 10.1128/AAC.01499-13 PMC3910754

[107] You X, Xiao Y, Liu K, et al Association of plasma antimony concentration with markers of liver function in Chinese adults. Environ Chem. 2020;17(4):304‐313.

[108] Xie Z, Aimuzi R, Si M, Qu Y, Jiang Y. Associations of metal mixtures with metabolic-associated fatty liver disease and non-alcoholic fatty liver disease: NHANES 2003-2018. Front Public Health. 2023;11:1133194. 36950101 10.3389/fpubh.2023.1133194 PMC10025549

[109] Zhong G, Wan F, Wu S, et al Arsenic or/and antimony induced mitophagy and apoptosis associated with metabolic abnormalities and oxidative stress in the liver of mice. Sci Total Environ. 2021;777:146082. 33676223 10.1016/j.scitotenv.2021.146082

[110] Soomro MH, England-Mason G, Liu Ji, et al Associations between the chemical exposome and pregnancy induced hypertension. Environ Res. 2023;237:116838. 37544468 10.1016/j.envres.2023.116838

[111] Vigeh M, Yokoyama K, Ramezanzadeh F, et al Lead and other trace metals in preeclampsia: a case-control study in Tehran, Iran. Environ Res. 2006;100(2):268‐275. 16029873 10.1016/j.envres.2005.05.005

[112] Wang Y, Wang K, Han T, et al Exposure to multiple metals and prevalence for preeclampsia in taiyuan, China. Environ Int. 2020;145:106098. 32916414 10.1016/j.envint.2020.106098

[113] Liu T, Zhang M, Rahman ML, et al Exposure to heavy metals and trace minerals in first trimester and maternal blood pressure change over gestation. Environ Int. 2021;153:106508. 33901931 10.1016/j.envint.2021.106508 PMC13138925

[114] Howe CG, Margetaki K, Vafeiadi M, et al Prenatal metal mixtures and child blood pressure in the rhea mother-child cohort in Greece. Environ Health. 2021;20(1):1. 33407552 10.1186/s12940-020-00685-9 PMC7789252

[115] Galvez-Fernandez M, Sanchez-Saez F, Domingo-Relloso A, et al Gene-environment interaction analysis of redox-related metals and genetic variants with plasma metabolic patterns in a general population from Spain: the hortega study. Redox Biol. 2022;52:102314. 35460952 10.1016/j.redox.2022.102314 PMC9048061

[116] Urbanowicz T, Hanć A, Frąckowiak J, et al Are hair scalp trace elements correlated with atherosclerosis location in coronary artery disease? Biol Trace Elem Res. 2025;203(4):2122‐2131. 39145863 10.1007/s12011-024-04335-w PMC11919964

[117] Guo L-C, Lv Z, Ma W, et al Contribution of heavy metals in PM2.5 to cardiovascular disease mortality risk, a case study in Guangzhou, China. Chemosphere. 2022;297:134102. 35219707 10.1016/j.chemosphere.2022.134102

[118] Cheng T, Yu D, Li G, Chen X, Zhou L, Wen Z. Association between exposure to urinary metal and all-cause and cardiovascular mortality in US adults. PLoS One. 2024;19(12):e0316045. 39729492 10.1371/journal.pone.0316045 PMC11676533

[119] Shi C, Zhi J, Zhao H, et al Risk of heavy metal(loid) compositions in fine particulate matter on acute cardiovascular mortality: a poisson analysis in Anyang, China. Int J Biometeorol. 2024;68(7):1275‐1286. 38625430 10.1007/s00484-024-02665-x

[120] Riffo-Campos AL, Fuentes-Trillo A, Tang WY, et al In silico epigenetics of metal exposure and subclinical atherosclerosis in middle aged men: pilot results from the Aragon Workers Health Study. Philos Trans R Soc Lond B Biol Sci. 2018;373(1748):20170084. 29685964 10.1098/rstb.2017.0084 PMC5915723

[121] Grau-Perez M, Caballero-Mateos MJ, Domingo-Relloso A, et al Toxic metals and subclinical atherosclerosis in carotid, femoral, and coronary vascular territories: the Aragon Workers Health Study. Arterioscler Thromb Vasc Biol. 2022;42(1):87‐99. 34879710 10.1161/ATVBAHA.121.316358

[122] Liu W, Yu L, Ye Z, et al Assessment for the associations of twenty-three metal(loid)s exposures with early cardiovascular damage among Chinese urban adults with five statistical methods: insight into assessing health effect of multipollutant exposure. Chemosphere. 2022;307:135969. 35940407 10.1016/j.chemosphere.2022.135969

[123] Phillips MA, Cánovas A, Rea MA, et al Deducing signaling pathways from parallel actions of arsenite and antimonite in human epidermal keratinocytes. Sci Rep. 2020;10(1):2890. 32076005 10.1038/s41598-020-59577-0 PMC7031270

[124] Tamás MJ . Cellular and molecular mechanisms of antimony transport, toxicity and resistance. Environ. Chem. 2016;13(6):955‐962.

[125] Zhou Y, Messier N, Ouellette M, Rosen BP, Mukhopadhyay R. Leishmania major LmACR2 is a pentavalent antimony reductase that confers sensitivity to the drug pentostam*. J Biol Chem. 2004;279(36):37445‐37451. 15220340 10.1074/jbc.M404383200

[126] Yan S, Wong ILK, Chow LMC, Sun H. Rapid reduction of pentavalent antimony by trypanothione: potential relevance to antimonial activation. Chem. Commun. 2003;2(2):266‐267. 10.1039/b210240d 12585423

[127] Zhang Y, Lv J, Sun X, et al Toxicity of antimony in housefly after whole-life-cycle exposure: changes in growth, development, redox homeostasis, mitochondrial function, and fecundity. Ecotoxicol Environ Saf. 2025;289:117656. 39752911 10.1016/j.ecoenv.2024.117656

[128] Sundar S, Chakravarty J. Antimony toxicity. Int J Environ Res Public Health. 2010;7(12):4267‐4277. 21318007 10.3390/ijerph7124267 PMC3037053

[129] Lai Z, He M, Lin C, Ouyang W, Liu X. Interactions of antimony with biomolecules and its effects on human health. Ecotoxicol Environ Saf. 2022;233:113317. 35182796 10.1016/j.ecoenv.2022.113317

[130] Asakura K, Satoh H, Chiba M, et al Genotoxicity studies of heavy metals: lead, bismuth, indium, silver and antimony. J Occup Health. 2009;51(6):498‐512. 19851040 10.1539/joh.l9080

[131] Litwin I, Mucha S, Pilarczyk E, Wysocki R, Maciaszczyk-Dziubinska E. Complex mechanisms of antimony genotoxicity in budding yeast involves replication and topoisomerase I-associated DNA lesions, telomere dysfunction and inhibition of DNA repair. Int J Mol Sci. 2021;22(9):4510. 33925940 10.3390/ijms22094510 PMC8123508

[132] Yao Q, Yang A, Hu X, et al Effects of antimony exposure on DNA damage and genome-wide variation in zebrafish (Danio rerio) liver. Aquatic Toxicology. 2023;259:106524. 37031539 10.1016/j.aquatox.2023.106524

[133] Cavallo D, Iavicoli I, Setini A, et al Genotoxic risk and oxidative DNA damage in workers exposed to antimony trioxide. Environ Mol Mutagen. 2002;40(3):184‐189. 12355552 10.1002/em.10102

[134] Alvarez M, Malécot CO, Gannier F, Lignon JM. Antimony-induced cardiomyopathy in Guinea-pig and protection by L-carnitine. Br J Pharmacol. 2005;144(1):17‐27. 15644865 10.1038/sj.bjp.0706030 PMC1575978

[135] Bento DB, de Souza B, Steckert AV, et al Oxidative stress in mice treated with antileishmanial meglumine antimoniate. Res Vet Sci. 2013;95(3):1134‐1141. 24012348 10.1016/j.rvsc.2013.08.004

[136] Tirmenstein MA, Plews PI, Walker CV, et al Antimony-induced oxidative stress and toxicity in cultured cardiac myocytes. Toxicol Appl Pharmacol. 1995;130(1):41‐47. 7839369 10.1006/taap.1995.1006

[137] Jiang X, Yu W, Wu S, et al Arsenic (III) and/or Antimony (III) induced disruption of calcium homeostasis and endoplasmic reticulum stress resulting in apoptosis in mice heart. Ecotoxicol Environ Saf. 2021;220:112394. 34091186 10.1016/j.ecoenv.2021.112394

[138] Tan Y, El-Kersh K, Watson SE, et al Cardiovascular effects of environmental metal antimony: redox dyshomeostasis as the key pathogenic driver. Antioxid Redox Signal. 2023;38(10-12):803‐823. 36424825 10.1089/ars.2022.0185 PMC10402706

[139] Kuryshev YA, Wang L, Wible BA, Wan X, Ficker E. Antimony-based antileishmanial compounds prolong the cardiac action potential by an increase in cardiac calcium currents. Mol Pharmacol. 2006;69(4):1216‐1225. 16418337 10.1124/mol.105.019281

[140] Choe S-Y, Kim S-J, Kim H-G, et al Evaluation of estrogenicity of major heavy metals. Sci Total Environ. 2003;312(1-3):15‐21. 12873394 10.1016/S0048-9697(03)00190-6

[141] Cen Z, Lv S, Li Q, et al Acute exposure to antimony elicits endocrine disturbances, leading to PCOS and ovarian fibrosis in female zebrafish. Comp Biochem Physiol C Toxicol Pharmacol. 2025;294:110198. 40174734 10.1016/j.cbpc.2025.110198

[142] Azhdari M, zur Hausen A. Wnt/β-catenin and notch signaling pathways in cardiovascular disease: mechanisms and therapeutics approaches. Pharmacol Res. 2025;211:107565. 39725339 10.1016/j.phrs.2024.107565

[143] Massagué J, Sheppard D. TGF-β signaling in health and disease. Cell. 2023;186(19):4007‐4037. 37714133 10.1016/j.cell.2023.07.036 PMC10772989

[144] Doke M, Avecilla V, Felty Q. Inhibitor of differentiation-3 and estrogenic endocrine disruptors: implications for susceptibility to obesity and metabolic disorders. Biomed Res Int. 2018;2018:6821601. 29507860 10.1155/2018/6821601 PMC5817379

[145] Belova A, Greco SL, Riederer AM, Olsho LEW, Corrales MA. A method to screen U.S. Environmental biomonitoring data for race/ethnicity and income-related disparity. Environ Health. 2013;12(1):114. 24354733 10.1186/1476-069X-12-114 PMC3893603

[146] Shiue I . People with diabetes, respiratory, liver or mental disorders, higher urinary antimony, bisphenol A, or pesticides had higher food insecurity: USA NHANES, 2005-2006. Environ Sci Pollut Res Int. 2016;23(1):198‐205. 26517997 10.1007/s11356-015-5677-y

[147] Barbieri FL, Gardon J, Ruiz-Castell M, et al Toxic trace elements in maternal and cord blood and social determinants in a bolivian mining city. Int J Environ Health Res. 2016;26(2):158‐174. 26179629 10.1080/09603123.2015.1061114 PMC4733940

[148] Geron M, Cowell W, Amarasiriwardena C, et al Racial/ethnic and neighborhood disparities in metals exposure during pregnancy in the Northeastern United States. Sci Total Environ. 2022;820:153249. 35065119 10.1016/j.scitotenv.2022.153249 PMC8930522

[149] Howe CG, Claus Henn B, Eckel SP, et al Prenatal metal mixtures and birth weight for gestational age in a predominately lower-income hispanic pregnancy cohort in Los Angeles. Environ Health Perspect. 2020;128(11):117001. 33141601 10.1289/EHP7201 PMC7608819

[150] Murphy M, Cohn D, Loria A. Developmental origins of cardiovascular disease: impact of early life stress in humans and rodents. Neurosci Biobehav Rev. 2017;74:453‐465. 27450581 10.1016/j.neubiorev.2016.07.018 PMC5250589

[151] Agency for Toxic Substances and Disease Registry. Antimony | Toxicological Profile | ATSDR. Accessed August 15, 2023. https://wwwn.cdc.gov/TSP/ToxProfiles/ToxProfiles.aspx?id=332&tid=58

[152] Barregard L, Ellingsen DG, Berlinger B, Weinbruch S, Harari F, Sallsten G. Normal variability of 22 elements in 24-hour urine samples—results from a biobank from healthy non-smoking adults. Int J Hyg Environ Health. 2021;233:113693. 33581414 10.1016/j.ijheh.2021.113693

